# Outcomes of multicystic peritoneal mesothelioma treatment with cytoreductive surgery and hyperthermic intraperitoneal chemotherapy

**DOI:** 10.1093/bjsopen/zraa001

**Published:** 2020-12-22

**Authors:** A Zahid, L Clarke, N Carr, K Chandrakumaran, A Tzivanakis, S Dayal, F Mohamed, T Cecil, B J Moran

**Affiliations:** Peritoneal Malignancy Institute, Basingstoke and North Hampshire Hospital, Basingstoke, UK

## Abstract

**Background:**

Multicystic peritoneal mesothelioma (MCPM) is a rare neoplasm, generally considered a borderline malignancy, best treated by cytoreductive surgery (CRS) to remove macroscopic disease, combined with hyperthermic intraperitoneal chemotherapy (HIPEC). Owing to its rarity, little has been published on clinical presentation, clinical behaviour over time, or an optimal treatment approach.

**Methods:**

A prospectively developed peritoneal malignancy database was interrogated for the years 2001–2018. Details on all patients with MCPM as a definitive diagnosis after CRS and HIPEC were analysed, including previous interventions, mode of presentation, surgical treatment, postoperative outcomes, and late follow-up information from abdominal CT and tumour markers.

**Results:**

Some 40 patients with MCPM underwent CRS and HIPEC between 2001 and 2018. Of these, 32 presented with abdominal pain, distension or bloating, six patients presented with recurrence following previous surgery at the referring hospitals, and two had coincidental diagnoses during a surgical procedure. CRS involved peritonectomy in all 40 patients. Bowel resection was required in 18 patients, and seven had a temporary stoma. Thirty-eight patients were considered to have undergone a complete macroscopic tumour removal (completeness of cytoreduction CC0), and two had residual tumour nodules less than 2.5 mm in size, classified as CC1. Median duration of follow-up was 65 (range 48–79) months. There were no deaths during follow-up. The Kaplan–Meier-predicted recurrence-free interval was 115.4 months.

**Conclusion:**

MCPM is a rare peritoneal neoplasm with a heterogeneous pattern of presentation. CRS and HIPEC is an effective management option for this group of patients, with favourable long-term survival.

## Resumen

**Antecedentes:**

El mesotelioma multiquístico (*multicystic mesothelioma*, MCPM) es una neoplasia peritoneal infrecuente, generalmente considerada como una neoplasia maligna limítrofe, que se trata mejor con cirugía citorreductora (*cytoreductive surgery*, CRS) para extirpar la enfermedad macroscópica combinada con quimioterapia intraperitoneal con hipertermia (*hyperthermic intraperitoneal chemotherapy*, HIPEC). Debido a su rareza, son muy pocas las publicaciones sobre su presentación clínica, comportamiento clínico a lo largo del seguimiento o abordaje terapéutico óptimo.

**Métodos:**

Se consultó una base de datos prospectiva de neoplasias malignas peritoneales durante el intervalo entre 2001 y 2018. Se analizaron los detalles de todos los pacientes con MCPM como diagnóstico definitivo tras CRS y HIPEC, incluyendo intervenciones previas, forma de presentación, tratamiento quirúrgico, resultados postoperatorios e información del seguimiento tardío del TC abdominal y marcadores tumorales.

**Resultados:**

Un total de 40 pacientes fueron sometidos a CRS y HIPEC por MCPM entre 2001 y 2018. De estos, 31 (77,%) consultaron inicialmente por dolor abdominal, distensión o hinchazón abdominal, 6 pacientes presentaban una recidiva después de una cirugía previa en los hospitales de referencia y en 2 pacientes el diagnóstico se estableció coincidiendo con una operación quirúrgica. La CRS incluyó peritonectomía en los 40 pacientes; 18/40 precisaron resección intestinal y a siete se les realizó un estoma temporal. En 38 pacientes se consideró que se había realizado una extirpación macroscópica completa del tumor (*complete macroscopic tumor removal*, CC0), mientras que 2 quedaron con nódulos tumorales residuales de un tamaño < 2,5 mm y fueron clasificados como CC1. La mediana de seguimiento fue de 65 meses (rango 48-79). No hubo fallecimientos en el periodo de seguimiento. El intervalo libre de recidiva previsto por el método de Kaplan Meier fue de 115,4 meses.

**Conclusión:**

MCPM es una neoplasia peritoneal infrecuente con un patrón en su presentación heterogéneo. CRS y HIPEC es una opción de tratamiento efectiva para este grupo de pacientes con supervivencia a largo plazo favorable.

## Introduction

Multicystic peritoneal mesothelioma (MCPM) is a rare primary mesothelial neoplasm. It has been reported predominantly to affect females in their reproductive years and accounts for 3–5 per cent of all abdominal mesotheliomas^1^. The features were first described macroscopically by Plaut, but the first histological description was by Mennemeyer and Smith in 1979, who defined the disease as ‘multicystic peritoneal mesothelioma’^2^. MCPM is characterized by multilocular peritoneal cysts, composed of mesothelial lining cells lining thin fibrous walls, most commonly occurring in the pelvis and other parts of the peritoneal cavity, although rare extraperitoneal locations have been described, such as pleural, spermatic cord, tunica vaginalis, and pericardium^3^. The aetiology is poorly understood. Unlike other variants of mesothelioma, there is no reported link to asbestos exposure^4^^,^^5^. Some have proposed that hormones, particularly oestrogen, may play a role, and hormone receptors have been found in some pathological specimens, although not in significant amounts^3^. An association with previous abdominal inflammation or surgery has been proposed by some, but not others^6^.

The clinical presentation of MCPM ranges from incidental findings on imaging, laparotomy or laparoscopy, to patients with abdominal pain, abdominal masses and, rarely, pneumoperitoneum^7^^,^^8^. Although historically termed benign multicystic mesothelioma, clinical experience suggests that MCPM is a borderline malignancy, in that recurrence is common unless treated adequately^9^. Although the cysts are generally slow-growing with gradual disease progression, there have been a few reports of malignant transformation; squamous metaplasia is not uncommon^10^^,^^11^.

Debulking of the cystic masses was the mainstay of treatment, but resulted in high recurrence rates of 40–50 per cent^1^. As a consequence, optimal treatment has been proposed to encompass a combination of cytoreductive surgery (CRS), along the principles of treatment for other peritoneal malignancies, combined with hyperthermic intraperitoneal chemotherapy (HIPEC), with reported recurrence rates of about 20 per cent^6^. Although operative mortality and major morbidity rates for CRS and HIPEC are low in specialized centres, recurrence rates despite this aggressive treatment, and the young age group affected, have led to debate as to whether this radical approach should constitute standard practice.

In addition, experience in treating MCPM is limited, such that patient counselling and consent are challenging, particularly in patients who are asymptomatic or have minimal symptoms.

The present series represents a single-centre experience in treating MCPM by CRS and HIPEC; clinical features, operative procedures, postoperative complications, and long-term follow-up are reported.

## Methods

Data on patients with histologically proven multicystic mesothelioma were extracted from a prospectively developed registry of patients with peritoneal malignancy who underwent surgery at the Peritoneal Malignancy Institute, Basingstoke Hospital, UK in 2001–2018. Annual follow-up is by CT of the abdomen and pelvis together with tumour markers, namely carcinoembryonic antigen (CEA), carbohydrate antigen (CA) 125 and CA19-9. Patients are generally followed up locally with transmission of results to Basingstoke, and telephone consolation or clinic review as necessary. Data census was at last clinic appointment. The local ethics committee considered the study as a service evaluation.

The standard referral recommendations were CT of the chest, abdomen and pelvis with oral and intravenous contrast, together with measurement of the three tumour markers, CEA, CA125 and CA19-9. A number of patients had imaging and other investigations at their local centre, some having had laparotomy or laparoscopy for previous surgical procedures or investigations, yielding a tissue diagnosis. Diagnostic laparoscopy was used for definitive assessment of the peritoneal cavity and a tissue diagnosis. The Peritoneal Cancer Index (PCI) was calculated to assess the extent of peritoneal cancer throughout 13 regions of the peritoneal cavity, with a score of 1–3 given for the burden of disease in each area^8–12^. Patients who proceeded to surgery underwent compete cytoreduction followed by HIPEC with either single- or double-agent chemotherapy for 60 min via an open abdomen technique. Any gastrointestinal tract anastomosis was performed after administration of HIPEC. Completeness of cytoreduction was documented by the CC scoring system, where CC0 represents no residual disease, CC1 indicates residual nodules up to 2.5 mm in size, and CC2 denotes as residual nodules larger than 2.5 mm. Patients were generally admitted to the ICU for 24 h, and subsequently managed on a dedicated ward in a protocolized postoperative recovery programme.

The database included information on patient demographics, clinical presentation, tumour marker (CEA, CA125 and CA19-9) levels, disease burden, operative details, pathological features, and outcome. The primary outcome measure was the time to recurrence, as defined on cross-sectional imaging (predominantly CT), and the need for further surgical intervention. Secondary outcome measures were the clinical presentation, number of patients requiring bowel resection with or without stoma formation, postoperative morbidity (Clavien–Dindo classification) and mortality. Supplementary details were obtained, where necessary, from patient’s clinical records.

### Statistical analysis

Continuous variables are presented as median (range) values, and categorical data as frequencies and percentages. Kaplan–Meier curves were plotted to determine the time to recurrence and survival outcomes, expressed as median values with 95 per cent confidence intervals. Statistical significance was analysed with the log rank (Mantel–Cox) test. Cox proportional hazards regression analysis was used to determine the hazard ratio (HR) of the survival distribution to the variables. *P <*0.050 was considered significant. Data were analysed using R (R Foundation for Statistical Computing, Vienna, Austria).

## Results

Between 2001 and 2018, of 2245 patients who had surgery for peritoneal malignancy, 102 had peritoneal mesothelioma. Of these, 40 (39.2 per cent) had CRS and HIPEC for multicystic mesothelioma.

Presenting features were mainly abdominal distension, bloating, and/or pain in 31 of the 40 patients. Six patients had progression of disease following surgery at the referring hospitals and two were diagnosed coincidentally during another surgical procedure (hysterectomy and inguinal hernia repair). One patient had CT-detected abnormalities 2 years after a right hemicolectomy for colonic cancer, initially considered to be colorectal peritoneal metastases. Systemic chemotherapy and non-progression of the abnormalities prompted referral and subsequent laparotomy, at which the only finding was multicystic mesothelioma.

Overall 26 of the 40 patients were women. The median age was 41.5 (range 21–69) years. Tumour markers were measured routinely; normal tumour marker ranges were 0–5 for CEA, 0–35 for CA125, and 0–33 for CA19-9. The median preoperative CEA level was 1 (range 0.5–3.9), and that for CA125 was 14 (2–138) μg/ml. Overall, seven patients had a high CA125 level; two of these patients developed recurrence. The median preoperative CA19-9 level was 6 (range 2.5–3814) kunits/l, with tumour marker elevation in two patients; neither of these two patients developed recurrence.

The median PCI was 8 (range 3–31). Overall, 38 of the 40 patients had CC0 cytoreduction; the remaining two patients were considered to have had a CC1 reduction. Resection of organs and peritonectomy procedures are summarized in *Table 1*.

**Table 1:** Organs removed and peritonectomy procedures

	No. of patients (*n*=40)
**Organs removed**	
Appendix	23
Small bowel (segment )	1
Right colon	9
Sigmoid colon	1
Rectum	7
Greater omentum	34
Lesser omentum	16
Gallbladder	18
Spleen	2
Right ovary	14
Left ovary	13
Uterus	12
**Pelvic mass**	2
**Peritonectomy**	
Pelvic	25
Right and left parietal	24
Left diaphragmatic	17
Right diaphgramatic	4
Right liver capsule	2

The chemotherapy used for HIPEC was doxorubicin and cisplatin in 25 patients and mitomycin C in 15.

Seven patients had a defunctioning ileostomy; all had the stoma reversed a median of 7 (range 3–11) months later. In one patient stoma closure was complicated by postoperative obstruction requiring laparotomy and small bowel resection with a prolonged hospital stay, including the need for parenteral nutritional support.

**Fig. 1:**
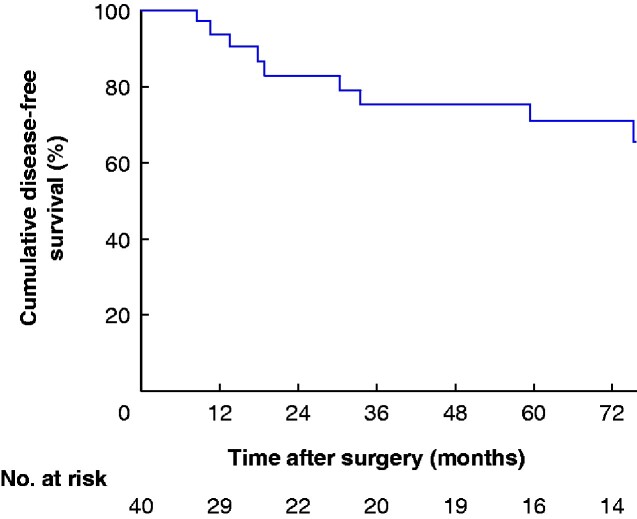
Kaplan–Meier-predicted recurrence-free survival of patients with multicystic peritoneal mesothelioma

The median duration of follow-up was 65.0 (95 per cent c.i. 48.4 to 79.0) months. There was no death during follow-up, and 25 patients had been alive for more than 5 years at the date of census. The mean Kaplan–Meier-predicted recurrence-free interval was 115.4 (95 per cent c.i. 93.8 to 137.0) months; the median was not reached (*Fig. 1*).

The mean predicted disease-free intervals for women and men were 132.6 (95 per cent c.i. 112.2 to 153.0) and 51.1 (30.0 to 72.1) months respectively. Cox regression analyses showed that only men had a significantly reduced disease-free interval with a hazard ratio of 6.0 (95 per cent c.i. 1.43 to 25.11; *P *=* *0.018) *versus* women. Other variables did not impact on the disease-free interval.

Thirty-five patients had Clavien–Dindo grade I or II complications. One patient had a grade IVa perioperative cardiac complication that necessitated readmission to the ICU, with complete resolution. One patient required emergency fasciotomy for a left lower-limb compartment syndrome on the night of surgery (grade IIIa complication). The overall rate of significant morbidity (grade III--IV) was thus 5 per cent (2 of 40).

During follow-up, two patients had surgical procedures: one had a laparotomy for small bowel obstruction 8 months after CRS, and one patient had multiple procedures for an unrelated cryptoglandular fistula *in ano*.

## Discussion

In line with other series, the most common presenting complaints in the present cohort were abdominal pain and non-specific abdominal symptoms^13^. Involvement of the greater omentum, parietal peritoneum, ovary, mesentery, and right iliac fossa were common features, again as reported elsewhere^14^. Six of the 40 patients in the present series had undergone previous surgery for cystic mesothelioma (1 patient had 3 previous operations between 1991 and 2000 with colovesical and colocutaneous fistulation after the third operation), indicating that recurrence and progression are not unusual events.

The increased female predominance of MCPM has been studied previously^3^, with no evidence of overexpression of oestrogen or progesterone receptors. Others have noted the high rate of recurrence after resection/debulking procedures. In one series^15^ this was approximately 50 per cent with a mean interval to recurrence of 32 months, and there have also been two case reports^16^^,^^17^ of malignant transformation. These results contrast markedly with those from the present series, where the mean recurrence-free interval was 115.4 months.

There is no standard treatment strategy for MCPM. In the past. many have advocated extensive surgical resection and debulking procedures^11^. Adjuvant systemic chemotherapy and/or radiotherapy have been described in addition to hormone therapy, sclerotherapy, and laser vaporization^10^^,^^18^. One patient in the present series initially underwent surgical excision in 1979 followed by chemotherapy and abdominal wall radiotherapy, presenting in 2010 with multicystic disease and a hard mass in the region of the caecum. A right hemicolectomy with CRS and HIPEC was performed. Histological findings were of a B-cell lymphoma treated by subsequent chemotherapy. CT in 2019 showed some stable low-volume cysts and no evidence of lymphoma.

Current literature suggests that operative intervention should be a combination of CRS and HIPEC, based on recurrence risk and potential for malignant transformation. In this series, this treatment approach resulted in a 5-year progression-free survival rate of more than 80 per cent, and a 10-year overall survival rate approaching 100 per cent. Published major postoperative complication rates range from 7 to 60 per cent after CRS and HIPEC, although these series included very extensive procedures for many patients with more aggressive and extensive peritoneal malignancy^1^^,^^6^. The practice in the present authors’ unit of removing all macroscopic disease before HIPEC without removal of macroscopically normal peritoneum resulted in a major complication rate of only 5 per cent.

The high risk of recurrence and potential for malignant transformation warrant consideration of surgical intervention for MCPM. The safety and efficacy of CRS and HIPEC in selected patients, performed in a unit specializing in peritoneal malignancy, support the continued use of this strategy as the preferred approach for this condition.

*Disclosure.* The authors declare no conflict of interest.
